# Discrimination Increases Suicidal Ideation in Black Adolescents Regardless of Ethnicity and Gender

**DOI:** 10.3390/bs7040075

**Published:** 2017-11-06

**Authors:** Shervin Assari, Maryam Moghani Lankarani, Cleopatra Howard Caldwell

**Affiliations:** 1 Department of Psychiatry, University of Michigan, Ann Arbor, MI 48109-2700, USA; lankaranii@yahoo.com; 2 Center for Research on Ethnicity, Culture, and Health, University of Michigan, Ann Arbor, MI 48109-2029, USA; cleoc@umich.edu; 3 Department of Health Behavior and Health Education, University of Michigan, Ann Arbor, MI 48109-2029, USA

**Keywords:** discrimination, mental health, suicidal ideation, Black youth

## Abstract

Background: Discrimination is a common experience for Blacks across various developmental periods. Although much is known about the effect of discrimination on suicidal ideation of adults, less is known about the same association in Black youth. Aim: We examined the association between discrimination and suicidal ideation in a national sample of Black youth. We also explored gender and ethnic differences in this association. Methods: We used data from the National Survey of American Life-Adolescents (NSAL-A), 2003–2004. In total, 1170 Black adolescents entered the study. This number was composed of 810 African American and 360 Caribbean Black youth (aged 13 to 17 years). Demographic and socioeconomic factors were controls, perceived discrimination was the predictor, and lifetime suicidal ideation was the outcome. Logistic regression was used to test the association between perceived discrimination and suicidal ideation in the pooled sample, as well as based on ethnicity and gender. Results: In the pooled sample of Black youth, higher perceived discrimination was associated with higher odds of suicidal ideation (Odds Ratio (OR) = 1.09; 95% Confidence Interval (CI) = 1.02−1.17). This association was significant net of age, ethnicity, gender, and socioeconomic status. We did not find interactions between perceived discrimination and ethnicity or gender on suicidal ideation. Perceived discrimination was associated with suicidal ideation in African Americans (CI = 1.09; 95% CI = 1.01−1.17) and Caribbean Blacks (CI = 1.16; 95% CI = 1.03−1.32), males (CI = 1.11; 95% CI = 1.00−1.25), and females (CI = 1.08; 95% CI = 1.00−1.16). Conclusion: Discrimination jeopardizes the mental health of Black youth. In a universal pattern, discrimination is associated with suicidal ideation in Black youth. More research is needed on this topic.

## 1. Background

The harmful effects of discrimination on the mental health of Black populations are well established [1,2,3]. However, less is known about the same effects in Black youth. The current study aims to examine the effect of discrimination on suicidal ideation in Black youth. This study also examines ethnicity and gender differences in the association between discrimination and suicidal ideation.

Discrimination is defined as differential and unjust treatment of individuals because of particular identities such as race, ethnicity, age, gender [4]. In the United States, Blacks experience high levels of discrimination, which increases the risk of undesired mental health outcomes such as psychological symptoms [5,6,7,8,9,10] and categorical psychiatric disorders [11]. A recent meta-analysis suggested that exposure to racial discrimination increases poor mental health outcomes for Blacks [11]. Similar to other stressors, the context in which discrimination happens has an important role in changing the effect of discrimination [12,13].

Although the negative effect of discrimination on the mental health of adults is well documented [2,11,14,15], fewer studies have focused on the negative influence of discrimination on the mental health of Black youth [16,17,18,19]. In Black youth, discrimination is linked to stress [19], low self-esteem [18,19], and psychiatric disorders [20]. Discrimination is also associated with depressive symptoms [18,19] as well as clinical depression [17].

Black youth may become more aware of their identities during their transition to adolescence and adulthood, and therefore may be sensitive to discriminatory experiences that relate to their racial and general identities [21,22]. As a result, discrimination experiences may become extremely important for the mental health and well-being of adolescents [23,24]. The negative impact of exposure to discrimination on the mental health of Blacks persists over time [17,25]. Cheng et al. [26] showed that discrimination in adolescence predicts future depression among Black youth. Sellers and Shelton have discussed how discrimination and identities interact among Black youth [27].

Gibbons et al. [28] showed that nine out of 10 Black adolescents report having had at least one experience of racial discrimination. Black adolescents experience discrimination across settings, including but not limited to school [29,30]. Although a wide range of mental health outcomes has been linked to discrimination, less is known about the effect of discrimination on suicide and Black youth.

Discrimination, however, may not have similar effects across all groups of Blacks. Hudson et al. [31] have shown that high discrimination may have stronger effects in the presence of high socioeconomic status (SES). Racial identity may also alter the effects of discrimination on mental health [32]. A number [27,33,34] but not all [10] studies have also shown that discrimination may have stronger effects for males than females, particularly in the presence of high masculine ideologies [35]. An interaction between ethnicity and gender has also been shown to alter these effects [3,10].

## 2. The Current Study

This study investigated the effects of discrimination, ethnicity, gender, and SES on suicidal ideation in a national sample of Black youth. We also tested gender and ethnic differences in this regard. We hypothesized that discrimination will be associated with more suicidal ideation for all groups of Black youth.

## 3. Method

### 3.1. Design and Setting

This is a cross-sectional study. We used data from the National Survey of American Life-Adolescent Supplement (NSAL-A), 2003 [9,36]. The NSAL was conducted as a part of the Collaborative Psychiatric Epidemiology Surveys (CPES) [37]. The NSAL-A is one of the largest national mental health surveys of Black youth in the United States [38].

### 3.2. Ethics

The University of Michigan Institute Review Board approved the NSAL study. Adolescents’ legal guardians provided informed written consent. All adolescents provided assent themselves. Respondents received $50 as financial compensation. The study was funded by the National Institute of Mental Health (NIMH) and was conducted by the University of Michigan, Ann Arbor, MI, USA.

### 3.3. Participants

The study enrolled 1170 Black adolescents, including 810 African Americans and 360 Caribbean Blacks. Participating youth ranged in age from 13 to 17 years. At the time of the study, all participants resided in the United States. More detailed information on the sampling strategy is available elsewhere [37,38].

### 3.4. Sampling

The NSAL-Adolescent sample was drawn from the NSAL, a national probability sample of adult Blacks in the United States. The NSAL-adult sample was screened for African American and Caribbean Black households with eligible adolescents living in the households. Adolescents living in households were randomly selected for participation. If more than one eligible adolescent lived in a household, two adolescents were selected based on the gender of the first eligible adolescent. This strategy resulted in non-independence for adolescent samples. In response to this lack of independence, the adolescent supplement data were weighted to adjust for non-independence of the selection probabilities and non-response at the household and individual levels. The weighted data were then post-stratified to represent national estimates based on gender, age, and ethnicity [9,10].

### 3.5. Interviews

All interviews were conducted in English. Interviews lasted 100 minutes on average. The overall response rate was 80.6%. The response rate was slightly higher for Caribbean Black youth (83.5%) than for African American youth (80.4%).

For the majority (82%) of the interviews, data collection occurred during in-person interviews conducted in the adolescents’ homes. The remaining 18% of interviews were conducted entirely or partially by telephone. All in-person interviews used computer-assisted personal interviews (CAPIs). With CAPI, computers are used by trained interviewers to conduct the interviews. CAPI is the preferred method of interviewing for long and complex questionnaires.

## 4. Measures

*Sociodemographics*: The study included demographic factors, such as age, gender, and ethnicity. Socioeconomic status (SES) was also measured. For SES, we used the poverty index, defined as the income-to-needs ratio from the 2001 United States Census. The poverty index was calculated by dividing household income by the poverty threshold [39]. Higher scores on the poverty index indicate higher SES.

*Ethnicity*: Ethnicity was the self-identified ethnicity of the family household in which the adolescent lived. Participants self-identified as either African Americans or Caribbean Blacks. African American was defined as Black without having ancestral ties to the Caribbean. Caribbean Black was defined as Black having ancestral ties to a country included on a list of Caribbean countries provided by the interviewer, or that the particpant’s parents or grandparents were born in a Caribbean country. Caribbean countries included Antigua and Barbuda, Barbados, Bahamas, Cuba, Dominican Republic, Dominica, Grenada, Haiti, Jamaica, Saint Vincent and the Grenadines, Trinidad and Tobago, Saint Lucia, and Saint Kitts and Nevis.

*Discrimination*: NSAL-A used a 13-item modified version of the Everyday Discrimination Scale (EDS) to measure discrimination. These items assess chronic, routine, and less overt discriminatory experiences that have occurred over the past year [40]. Although the original measure includes 10 items, NSAL-A has added three additional items that reflect perceived teacher discrimination. Although this measure was originally developed and normalized among adults, it also operates well for adolescents [9,40,41]. Respondents were asked: “In your day-to-day life, how often have any of the following things happened to you?” Sample items include: “being followed around in stores”, “people acting as if they think you are dishonest”, “receiving poorer service than other people at restaurants”, and “being called names or insulted”. The Likert response scale ranged from 1 (never) to 6 (almost every day). A sum score was calculated, reflecting the frequency of exposure to discriminatory events over the past year (α = 0.86).

*Suicidal ideation*: Lifetime serious suicidal ideation was asked using the following single-item measure: Did the following experience ever happen to you? “You seriously thought about killing yourself”. Single-item measures of suicidal ideation have been frequently used in large-scale national epidemiological studies [34,42,43,44]. Only 11.3% of participants that endorse a single-item suicide attempt engage in behavior that would not meet the standard definition of a suicide attempt. Similarly, only 8.8% of individuals who endorse a single-item measure of suicidal ideation endorse thoughts that would not meet standard definitions of suicidal ideation. These are indicative of an acceptable validity of a single-item measure of suicidal ideation [45].

## 5. Statistical Analysis

To accommodate the complex design of the NSAL-A, Stata 13.0 (Stata Corp., College Station, TX, USA) was used for data analysis. Adjusted odds ratios (ORs) and their 95% confidence intervals were reported.

We used the Taylor expansion approximation technique to re-calculate the complex design-based estimates of variance. Standard errors reflect the weights due to the complex sampling design. All percentages reported in this study are weighted. As the Caribbean Black sample is more clustered than the African American sample, the standard errors are systematically larger for Caribbean Black than that for African American youth. As a result, findings are more conservative for Caribbean Black youth.

We used survey logistic regression for multivariable analysis. In our model, discrimination was the main predictor, lifetime suicidal ideation was the main outcome, and age, gender, ethnicity, and the poverty index were covariates. We fit multiple logistic regression models to determine the role of discrimination on suicidal ideation in the pooled sample as the subgroups. In the first step, the association of interest was estimated in the pooled sample. In the next step, we added the interaction terms (between discrimination and ethnicity or gender) to the model. Finally, we ran models specific to ethnicity and gender.

From logistic regressions, we reported Odds Ratios (ORs) and associated 95% Confidence Intervals (CIs). *p* values less than 0.05 were considered statistically significant. Missing data was not imputed. We used complete case analysis for our regression models.

## 6. Results

### 6.1. Descriptive Statistics

The sample was evenly composed of boys (*n* = 563, 48%) and girls (*n* = 605, 52%). The mean age of the participants was 15 years (SD *=* 1.42). Of the sample, 40% were between the ages of 13 and 14 years (*n* = 477), 41% were between the ages 15 and 16 years (*n* = 441), and 19% were age 17 years (*n* = 252). Most (96%) participants were enrolled in high school. The median family income was US$28,000, with a range from 0 to US$520,000.

### 6.2. Bivariate Associations

Caribbean Blacks (US$32,250) had a higher median income than African Americans (US$26,000) (*p* < 0.001). Caribbean Blacks were also older than African Americans (*p* < 0.05). The proportion of girls was marginally higher for Caribbean Blacks than African Americans (*p* < 0.1). African Americans and Caribbean Blacks reported similar levels of discrimination. However, boys reported significantly higher levels of discrimination compared to girls (*p* < 0.05). African Americans and Caribbean Blacks did not differ in suicidal ideation (*p* = 0.586). Suicidal ideation was more common among girls than boys (OR = 1.79; 95% CI = 1.01–3.16, *p* = 0.045) (Table 1).

### 6.3. Logistic Regression Models in the Pooled Sample

Table 2 summarizes the results of four logistic regressions in the pooled sample, with suicidal ideation as the outcome, discrimination as the predictor, and age, gender, ethnicity, and the poverty index as covariates. *Model 1* only included the main effects. In *Model 2*, we included an interaction term between ethnicity and discrimination. *Model 3* included an interaction term between gender and discrimination. *Model 4* included both interaction terms (Table 2).

*Model 1* in the pooled sample of Black youth showed that higher perceived discrimination was associated with higher odds of suicidal ideation (OR = 1.09; 95% CI = 1.02–1.17). This association was significant above and beyond age, ethnicity, gender, and socioeconomic status. Models 2 to 4 did not find any interactions between ethnicity or gender with perceived discrimination on suicidal ideation (Table 2).

### 6.4. Logistic Regression Models Based on Ethnicity and Gender

Table 3 presents the results of four logistic regression models with discrimination as the predictor and suicidal ideation as the outcome in ethnic and gender groups. Perceived discrimination was associated with suicidal ideation in African Americans (CI = 1.09; 95% CI = 1.01–1.17), Caribbean Blacks (CI = 1.16; 95% CI = 1.03–1.32), males (CI = 1.11; 95% CI = 1.00–1.25), and females (CI = 1.08; 95% CI = 1.00–1.16) (Table 3).

## 7. Discussion

The current study found an association between discrimination and suicidal ideation in a national sample of Black youth. Discrimination was a universal risk factor for suicidal ideation among Black youth, regardless of their ethnicity or gender.

The effect of discrimination as a risk factor for suicidal ideation among Black youth is in line with research showing that discrimination jeopardizes a wide range of mental health outcomes for adolescents [11] as well as adults [40,41]. In a recent study, we found that discrimination predicted the deterioration of the mental health of Blacks decades later [46].

We did not find gender differences in the association between discrimination and suicidal ideation of Black youth. At least three previous studies have shown the stronger effects of discrimination on undesired mental health outcomes for males than females [27,46,47]. Discrimination was particularly detrimental for Black males who had high SES [31,48] or high masculinity ideologies [35].

The differences observed between the results of this study and previous research on gender differences in the link between discrimination and suicide may be in part due to sampling (national datasets versus local sample), study setting (clinical versus community studies), or developmental age (early, mid, late adolescents, and emerging adults). It is not clear whether gender differences in suicidal ideation become more significant during emerging adulthood or not. It is also likely that gender differences are more pronounced for mental health outcomes rather than suicidal ideation.

Our study showed gender differences in exposure to discrimination, with males reporting more discrimination than females. There are other studies showing that among Blacks, males report higher rates of perceived discrimination compared to females [49,50]. Such gender differences in discrimination may be due to measurement or due to salience of dominance and hierarchy for males [51]. Discrimination may be more common among Black men [52] as they also experience mass incarceration and police brutality [53]. In addition, Black men may be more prone to its effects [51]. In a recent study of Black men, discrimination fully mediated the effect of incarceration history on poor mental health outcomes [16].

The current study did not find any ethnic differences in the effects of discrimination on suicidal ideation among Black male and female youth. These results are different from the previous studies that have shown ethnic differences in mental health effects of discrimination among Blacks. In the first study, Seaton et al. [9] reported a three-way interaction between ethnicity, gender, and discrimination on the mental health outcomes of Black youth. Specifically, the effects of perceived discrimination on depressive symptoms and life satisfaction were stronger for Caribbean Black females compared to African American males. In the second study using a national sample of Black adults, Assari, Watkins, and Caldwell [3] found a three-way interaction between gender, ethnicity, and race attribution on clinical depression. The study showed the strongest effect of discrimination on depression among Caribbean Black males who did not see race as a major barrier against upward social mobility. Ethnicity may alter susceptibility to discrimination through ethnic identity [32], social class [31], and expectations [54].

Although we found that females reported more suicidal ideation than males and males reported more discrimination than females in the current study, we did not find the same ethnicity by gender differences in the effects of discrimination on suicidal ideation as other studies with different mental health outcomes. This finding suggests that the nature of suicidal ideation in the face of discrimination is equally harmful for all groups of Black youth.

## 8. Limitations

This study had a few limitations. First, given the cross-sectional design of the study, the associations reported here should not be interpreted as causation. Second, the sample size was not identical across gender and ethnic groups. Third, the main outcome was evaluated using a single-item measure. Although this is a common approach in studying suicidal ideation in large-scale national studies [34,42,43,44], more comprehensive measures provide results with higher validity [55]. Fourth, a wide range of unmeasured factors such as negative life events, history of trauma and abuse, family support, psychiatric disorders, and access to healthcare may confound the results. Religious involvement, family support, ethnic identity, race-related attitudes, and attribution system may all alter the mental health consequences of discrimination.

## 9. Future Directions

Future research might delineate ways to focus on protective factors as well as effective interventions that can undo racism and discrimination. Additional research is needed with a more comprehensive set of control variables. A host of variables may contribute to the observed association between experiences of discrimination and suicidal ideation. For example, negative affectivity, neuroticism, or vigilance could be third variables that contribute to both perceived discrimination and suicidal ideation. There is also a need for studies that delve into mechanisms that link perceived discrimination to suicidal ideation. Furthermore, there is a need to study higher levels and structural factors that shape the mental health of Black youth. Research should also test whether interventions are similarly effective for different gender and ethnic groups.

## 10. Conclusions

In summary, in line with the literature that shows that discrimination jeopardizes other mental health outcomes, we found that discrimination was associated with suicidal ideation in Black youth. This association was found to be universal regardless of the ethnicity or gender of the youth.

## Figures and Tables

**Table 1:** Descriptive statistics of the sample overall, and based on ethnicity and gender.

	All	African Americans	Caribbean Blacks	Males	Females
	% (SE)	% (SE)	% (SE)	% (SE)	% (SE)
Age	14.95 (0.06)	14.92 (0.06) *	15.27 (0.04)	14.96 (0.06)	14.93 (0.09)
Poverty Index	4.05 (0.12)	4.03 (0.13)	4.25 (0.17)	4.02 (0.18)	4.07 (0.11)
Discrimination	5.04 (0.22)	5.04 (0.23)	5.02 (0.40)	5.31 (0.29) *	4.78 (0.22)
	% (95% CI)	% (95% CI)	% (95% CI)	% (95% CI)	% (95% CI)
Gender					
Male	50.05 (46.51–53.59)	50.42 * (46.59–54.25)	44.78 (39.98–49.68)	-	-
Female	49.95 (46.41–53.49)	49.58 (45.75–53.41)	55.22 (50.32–60.02)	-	-
Ethnicity					
African Americans	93.37 (91.88–94.60)	-	-	94.07 * (92.69–95.20)	92.67 (90.63–94.30)
Caribbean Blacks	6.63 (5.40–8.12)	-	-	5.93 (4.80–7.31)	7.33 (5.70–9.37)
Suicidal Ideation					
No	92.40 (90.28–94.09)	92.51 (90.23–94.30)	90.89 (82.11–95.59)	94.41 (90.99–96.58)	90.42 (87.52–92.70)
Yes	7.60 (5.91–9.72)	7.49 (5.70–9.77)	9.11 (4.41–17.89)	5.59 (3.42–9.01)	9.58 (7.30–12.48)

* *p* < 0.1; M: Mean; SE: Standard Error.

**Table 2:** Summary of logistic regressions for the associations between discrimination and suicidal ideation in the sample overall.

	*Model 1*		*Model 2*		*Model 3*		*Model 4*	
	OR	95% CI	OR	95% CI	OR	95% CI	OR	95% CI
Age	1.24 **	1.07–1.43	1.23 *	1.06–1.43	1.23 **	1.07–1.44	1.23 **	1.06–1.43
Ethnicity (Caribbean Black)	1.07	0.52–2.21	1.07	0.52–2.19	1.07	0.34–2.30	0.88	0.34–2.29
Gender (Female)	2.03 *	1.08–3.82	2.48	0.77–7.93	2.03 *	1.08–3.82	2.48	0.78–7.96
Poverty Index	1.05	0.90–1.24	1.05	0.90–1.24	1.05	0.90–1.24	1.05 #	0.90–1.16
Discrimination	1.09 *	1.02–1.17	1.08 *	1.00–1.16	1.09 *	1.02–1.17	1.08 *	1.00–1.17
Discrimination × Gender	-	-	1.03	0.90–1.18	-	-	1.03	0.90–1.18
Discrimination × Ethnicity	-	-	-	-	1.03	0.93–1.15	1.03	0.93–1.15

# *p* < 0.10, * *p* < 0.05, ** *p* < 0.001. OR; Odds Ratio. SE; Standard Error. CI; Confidence Interval.

**Table 3:** Summary of logistic regressions for the associations between discrimination and suicidal ideation based on ethnicity and gender.

	Ethnicity				Gender			
	African Americans		Caribbean Blacks		Male		Female	
	OR	95% CI	OR	95% CI	OR	95% CI	OR	95% CI
Age	1.22 **	1.07–1.41	1.20	0.41–3.51	1.25 *	0.91–1.72	1.20	0.95–1.52
Ethnicity (Caribbean Black)					0.34	0.10–1.23	1.48	0.60–3.64
Gender (Female)	1.87 #	0.97–3.61	9.65	1.81–51.45				
Poverty Index	1.04	0.88–1.24	1.21	0.77–1.91	1.07	0.82–1.40	1.04	0.83–1.31
Discrimination	1.09 *	1.01–1.17	1.16 *	1.03–1.32	1.11 *	1.00–1.25	1.08 *	1.00–1.16

# *p* < 0.10, * *p* < 0.05, ** *p* < 0.001. OR: Odds Ratio. SE: Standard Error. CI: Confidence Interval.
